# *Hdac4* Regulates the Proliferation of Neural Crest-Derived Osteoblasts During Murine Craniofacial Development

**DOI:** 10.3389/fphys.2022.819619

**Published:** 2022-02-15

**Authors:** Nayoung Ha, Jian Sun, Qian Bian, Dandan Wu, Xudong Wang

**Affiliations:** ^1^Department of Oral and Craniomaxillofacial Surgery, Shanghai Ninth People’s Hospital, Shanghai Jiao Tong University School of Medicine, Shanghai, China; ^2^National Clinical Research Center for Oral Diseases, Shanghai Key Laboratory of Stomatology, Shanghai Research Institute of Stomatology, Shanghai, China; ^3^Shanghai Institute of Precision Medicine, Shanghai, China

**Keywords:** HDAC4, neural crest, craniofacial skeletal development, frontal bone, cell proliferation

## Abstract

Craniofacial development involves the regulation of a compendium of transcription factors, signaling molecules, and epigenetic regulators. Histone deacetylases (HDACs) are involved in the regulation of cell proliferation, differentiation, and homeostasis across a wide range of tissues, including the brain and the cardiovascular, muscular, and skeletal systems. However, the functional role of *Hdac4* during craniofacial development remains unclear. In this study, we investigated the effects of knocking out *Hdac4* on craniofacial skeletal development by conditionally disrupting the *Hdac4* gene in cranial neural crest cells (CNCCs) using Cre-mediated recombination. Mice deficient for *Hdac4* in CNCC-derived osteoblasts demonstrated a dramatic decrease in frontal bone formation. *In vitro*, pre-osteoblasts (MC3T3-E1 cells) lacking *Hdac4* exhibited reduced proliferative activity in association with the dysregulation of cell cycle-related genes. These findings suggested that *Hdac4* acts, at least in part, as a regulator of craniofacial skeletal development by positively regulating the proliferation of CNCC-derived osteoblasts.

## Introduction

In vertebrates, the formation of a properly developed head is a complex process that requires the coordinated integration of multiple signals from the different germ layers (ectoderm, mesoderm, and endoderm) and their derivatives in concert with the precise regulation of cell migration, proliferation, and differentiation. Craniofacial development begins when cranial neural crest cells (CNCCs), a vertebrate-specific cell population, undergo dorsoventral migration. When migratory CNCCs reach the pharyngeal arches, they begin to proliferate, subsequently giving rise to a variety of head structures, including the craniofacial skeleton, craniofacial nerves, periodontal ligament, and dental mesenchyme (Depew et al., 2002). The specification, migration, and differentiation of CNCCs is regulated by a multitude of transcription factors, signaling molecules, and epigenetic regulators.

Histone deacetylases (HDACs) are key epigenetic enzymes that function by removing acetyl groups from core histones or non-histone proteins, thereby promoting chromatin condensation and regulating transcription. HDACs are characterized as Class I (HDAC1, 2, 3, and 8), and Class II (HDAC4, 5, 6, 7, 9, and 10) based on their protein structure, function, and subcellular localization. HDACs are involved in the regulation of cell proliferation, differentiation, and homeostasis across a wide range of tissues, including the brain and the cardiovascular, muscular, and skeletal systems. Because of their critical functions in gene regulation, HDACs are among the most extensively investigated drug targets, and HDAC inhibitors have been approved for the treatment of many human diseases, including cancer (Duvic and Vu, 2007). However, HDAC inhibitor activity has been implicated in abnormal head development. Notably, valproate syndrome has been described in children born to mothers treated with the HDAC inhibitor valproic acid. The facial features associated with valproate syndrome include a tall forehead with bifrontal narrowing, a flat nasal bridge, and philtrum flattening (Alsdorf and Wyszynski, 2005; Clayton-Smith et al., 2019), strongly suggesting that HDACs play a critical role in craniofacial development and morphogenesis.

In line with these teratogenic effects of HDAC inhibitors, the knockout of several class I HDACs leads to craniofacial abnormalities (Menegola et al., 2006; Haberland et al., 2009; Singh et al., 2013). The conditional deletion of *Hdac3* in CNCCs in the mouse results in cleft palate, microcephaly, frontal bone defects, and improper formation of the zygomatic arch due to enhanced expression of *Msx1*/2 and increased apoptosis of CNCCs (Singh et al., 2013). Similarly, conditional *Hdac8* deletion in CNCCs impairs calvarial development by negatively regulating the expression of *Otx2* and *Lhx1* (Haberland et al., 2009). These findings suggest that different HDAC members may play distinct roles in regulating the development of different parts of craniofacial structures. However, the functions of other HDACs, particularly the class II HDACs, during craniofacial development are less well understood.

Histone deacetylase 4 (HDAC4), a member of the class IIa group of HDACs, has been linked with craniofacial development. In humans, HDAC4 haploinsufficiency leads to brachydactyly mental retardation syndrome (BDMR, OMIM: 600430), also known as 2q37 deletion syndrome, characterized by craniofacial abnormalities, including a prominent forehead, midface retrusion, microcephaly, and a depressed nasal bridge (Williams et al., 2010). High-throughput SNP genotyping and gene expression analysis of infants born with cleft lip and/or palate and their parents showed that HDAC4 is a highly associated candidate gene for BDMR (Park et al., 2006). Additionally, the loss of *hdac4* function in zebrafish leads to impaired palate formation and a shortened face (DeLaurier et al., 2012, 2019). However, the role of *Hdac4* in murine craniofacial development has not been explicitly investigated.

Several studies have suggested that *Hdac4* plays an important role in skeletal development. *Hdac4* has been demonstrated to be an important regulator of chondrocyte maturation and initiation of endochondral ossification in mice (Vega et al., 2004). *Hdac4* suppresses chondrocyte hypertrophy and endochondral bone formation by binding to and suppressing the transactivation of *Runx2* and *Mef2C*. Mice lacking *Hdac4* display early chondrocyte maturation, resulting in ectopic bone formation. The conditional knockout of *Hdac4* in mouse osteoblasts using *Col2.3aI-Cre* influences cortical bone mass and thickness and leads to smaller stature, including short and stiff tails (Nakatani et al., 2018). Meanwhile, overexpressing *Hdac4* in proliferating chondrocytes inhibits bone formation in the process of endochondral ossification (Vega et al., 2004). However, most CNCC-derived craniofacial skeletal tissues undergo intramembranous ossification, in contrast to the endochondral ossification seen in skeletal tissues in other locations of the body. Intramembranous ossification consists of various stages, from cell proliferation and differentiation to matrix maturation and mineralization. No report to date has described intramembranous ossification defects during murine craniofacial skeleton development associated with changes in the expression of any class II HDAC. Consequently, whether and how *Hdac4* influences craniofacial skeleton development remains unknown.

Based on previous findings regarding the critical role of *Hdac4* in murine skeletal development and the proliferation of various cell types, the aim of this study was to evaluate the functional significance of *Hdac4* in craniofacial skeletal development. We found that *Hdac4* is expressed during craniofacial skeletal development in the mouse. The fact that expression of *Hdac4* is detected in osteogenic sites from embryonic day 14.5 (E14.5) suggested that *Hdac4* participates in craniofacial skeletal development. Because the craniofacial deformities in valproate syndrome caused by HDAC inhibition mainly occur in frontal bones, we focused on the role of *Hdac4* in frontal bone development. Using conditional ablation of *Hdac4* in CNCCs, we found that craniofacial skeletal development, particularly frontal bone development, is sensitive to *Hdac4* expression levels. *Hdac4*^fl/fl^*;Wnt1-Cre* mice exhibited reduced cell proliferation in developing frontal bone, leading to a reduction in frontal bone formation. *In vitro*, the knockdown of *Hdac4* in the pre-osteoblastic cell line MC3T3-E1 led to the dysregulation of cell cycle-related genes, such as *Cdkn1a*, *Cdk1*, and *Pcna*, resulting in subsequent cell cycle arrest at the G1 phase. Together, our data suggest that *Hdac4* plays a critical role in the regulation of cell proliferation during craniofacial skeletal development. Our findings not only improve the understanding of the intricate regulatory network underlying craniofacial development, but also provide critical information for interpreting the teratogenic effects of HDAC-targeted drugs.

## Materials and Methods

### Animals

*Wnt1-Cre* mice were obtained from the Jackson Laboratory and crossed with *Hdac4^fl/fl^* mice. In all timed pregnancies, the day of the appearance of a vaginal plug was defined as E0.5. P0 pups and embryos were collected for serial examination. For embryo harvesting, pregnant female mice were sacrificed by CO_2_ intoxication. The gravid uterus was dissected out and suspended in cold PBS. The embryos were harvested after amnionectomy and removal of the placenta. Embryos at the E12.5, E14.5, E15.5, and E16.5 stages (12:00 h of the day when the vaginal plug was detected was counted as E0.5) and P0 pups were used for subsequent experiments. All animal experiments were approved by the Animal Experimental Ethical Inspection of the Shanghai Ninth People’s Hospital affiliated to Shanghai Jiao Tong University, School of Medicine.

### Skeletal Preparation

Cartilage and bone in whole mouse embryos and pups were visualized after the clarification of soft tissue with potassium hydroxide and staining with Alcian Blue and Alizarin Red S (Sigma-Aldrich, St. Louis, MO, United States) as previously described (Nakashima et al., 2002; Ho et al., 2015; Parada and Chai, 2015).

### Micro-Computed Tomographic Imaging and 3D Reconstruction

Micro-computed tomography (micro-CT) was performed using a SkyScan 1176 (Bruker, Germany). Micro-CT images were acquired from P0 mice with the X-ray source voltage of 45 kV and current of 550 μA. Data were collected at a resolution of 18 μm. Volume rendering in 3D was achieved using Mimics (Materialize). Micro-CT scans of three replicates per genotype per stage were evaluated. All landmarks were determined based on Landmark Viewer (Richtsmeier Laboratory, Pennsylvania State University) available at www.getahead.la.psu.edu.

All the bones used in this study were manually segmented. Micro-CT scanning data were uploaded to Mimics as DICOM files. The background noise from these segmentations and bones outside the scope of this study were manually removed. The remaining craniofacial bones were isolated and labeled using pre-scale thresholds. Volumetric data were then rendered using Mimics’s 3D calculation tools and 3-matic (Materialize) was used for the measurements of isolated bones. The mean measurements of the bones were compared between the P0 control and mutant groups.

### Histology, Immunohistochemistry, and Immunofluorescence

For histology and immunofluorescence, the heads of the pups and embryos were fixed overnight in 4% PFA, dehydrated to 100% ethanol, embedded in paraffin, and sectioned into 5-μm-thick slices. Immunohistochemical and immunofluorescence staining was performed with anti-Hdac4 polyclonal antibody (ab12172, Abcam, United States, 1:250), anti-Runx2 antibody (sc-390351, Santa Cruz, United States, 1:50), anti-Ki67 rabbit monoclonal antibody (ab16667, Abcam, 1:200), anti-P21/Cdkn1a mouse monoclonal antibody (sc-6246, Santa Cruz, 1:50), goat polyclonal secondary antibody to rabbit IgG (ab150078, Abcam, 1:500), and goat polyclonal secondary antibody to mouse IgG (ab150114, Abcam, 1:500) following a previously described protocol (Zhao et al., 2020). Images were captured using an Olympus IX83 inverted microscope.

### Cell Culture

MC3T3-E1 cells were purchased from the Cell Bank of the Chinese Academy of Sciences, Shanghai, and maintained at 37°C with 5% CO_2_. The cells were cultured in MEM-α containing 10% FBS and 1% penicillin/streptomycin. For tasquinimod treatment, the cells were treated with tasquinimod (10 μM) for 7 days.

The culture medium was changed every 2 days.

### Construction of Short Hairpin RNA Expression Vectors and Cell Infection

Three short hairpin RNAs (shRNAs) targeting Hdac4 (NC_000067; Hdac4-shRNA1, GCAGATCCAGCGGCAGATACT; Hdac4-shRNA2, GCAGTTGTCCCGACAGCATGA; Hdac4-shRNA3, GCATGAGGCACAGTTGCATGA) and scrambled non-silencing RNA (TTCTCCGAACGTGTCACGT) were designed and synthesized by Genomeditech (Shanghai, China). The shRNAs were cloned into the pGMLV-HU6-MCS-CMV-ZsGreen1-PGK-Puro lentiviral vector yielding recombinant lentiviral shRNA expression vectors. ShRNA-containing lentiviruses were packaged and amplified by co-transfecting pGMLV-HU6-MCS-CMV-ZsGreen1-PGK-Puro vector together with pSPAX2 and pMD2G into 293T cells (at 70–80% confluence) in the presence of Lipofectamine 2000. At 48 h, the supernatants were collected and filtered through 0.22-μm cellulose acetate filters (Millipore, Billerica, MA, United States). The viral titer was determined by endpoint dilution assay. MC3T3-E1 cell suspensions were generated by trypsinization. The suspensions were placed in six-well plates at 2 × 10^4^ cells/well, incubated at 37°C with 5% CO_2_ for 24 h, and then infected with shRNA-lentiviruses (5 μg/mL) in polybrene (Beyotime Institute of Biotechnology, Jiangsu, China) for 12 h. Following the addition of normal culture medium, the cells were incubated for a further 48 h. To obtain stably infected cells, MC3T3-E1 cells were cultured in MEM-α supplemented with 5 μg/mL puromycin (Beyotime Institute of Biotechnology) for 7 days.

### EdU Assay

Cell proliferation was assayed using a BeyoClick™ EdU-555 Imaging Kit (Beyotime Institute of Biotechnology) according to the manufacturer’s instructions. Cells were incubated in the presence of 10 μM EdU for 2 h after which azide 555 was added to detect the incorporated EdU. The EdU-positive nuclei were counted in at least three randomly selected fields of view on microscopic images taken at ×100 and ×200 magnification using an Olympus IX83 inverted microscope. EdU-positive cells were quantified using ImageJ software (NIH).

### Real-Time Reverse Transcription-Quantitative PCR

For RT-qPCR analysis, 1 μg of total RNA was reverse transcribed into cDNA using Hifair 1*^st^* Strand cDNA Synthesis SuperMix for qPCR (11141ES10; Yeasen Biotech, China) following the manufacturer’s instructions. Quantitative PCR was performed on a Lightcycler 96 (Roche) with Hieff qPCR SYBR Green Master Mix (No Rox) (11201ES03; Yeasen Biotech). Relative mRNA expression levels were calculated using the 2^–ΔΔCT^ method and normalized to those of *GAPDH*. The sequences of all the primers used in this study are shown in Supplementary Table 1.

### Western Blotting

For Western blot analysis, total protein was extracted from cultured cells in protein lysis buffer (50 mM Tris–HCl, pH 8.0; 10 mM NaCl; 10% NP-40) containing protease and phosphatase inhibitors (P1045, Beyotime Institute of Biotechnology) on ice. Equal amounts of protein (40 μg/lane) were separated by (10%) SDS–PAGE (120 V) and transferred to polyvinylidene fluoride membranes (FFP24, Beyotime Institute of Biotechnology; 100 V for 120 min), and then incubated first with antibodies against Hdac4 (mouse monoclonal; Cell Signaling Technology, #7628, 1:2,000), Pcna (mouse monoclonal; Cell Signaling Technology, #2586, 1: 2,000), and β-actin (rabbit monoclonal; Cell Signaling Technology, #4970, 1:1,000) and then with HRP-conjugated goat anti-rabbit secondary antibody (LF102, Epizyme, China) and HRP-conjugated goat anti-mouse secondary antibody (LF101, Epizyme). The membranes were washed and bound antibodies were visualized using a chemiluminescence reagent (P0018A, Beyotime Institute of Biotechnology).

### Cell Counting Kit-8 Assay

Cells were plated in 96-well plates (1,000 cells/well) and incubated at 37°C with 5% CO_2_ for 24 h. Cell counting kit-8 (CCK-8) reagent (10 μL; Yeasen Biotech) mixed with 100 μL of mesenchymal stem cell medium was then added to each plate followed by incubation at 37°C with 5% CO_2_ for 1 h. Cell proliferation was assessed by measuring the optical density (OD) using an Epoch microplate reader (BioTek, United States).

### Cell Cycle Analysis by Flow Cytometry

Cells were plated in 100-mm diameter dishes at a 1:3 dilution and incubated at 37°C with 5% CO_2_ for 24 h. The cells were then rinsed with sterile PBS, harvested using 0.25% trypsin-EDTA, resuspended in PBS, centrifuged at 1,000 rpm for 5 min at room temperature (RT), washed twice with PBS, and fixed in chilled 70% ethanol overnight at −20°C. The next day, the cells were washed with PBS, treated with RNase (100 μg/mL), stained with propidium iodide (50 μg/mL) for 30 min at RT, and subjected to flow cytometry using a BD LSRFortessa system (BD Biosciences, San Jose, CA, United States). Data were analyzed using FlowJo (FlowJo LLC) software.

### Statistical Analysis

GraphPad Prism v.7.0a for Windows (GraphPad Software, La Jolla, CA, United States) was used for the statistical analysis. All numerical data are presented as means ± SD. Independent two-tailed Student’s *t*-tests were used for comparisons between two groups. Differences were considered statistically significant at *p*-value < 0.05.

## Results

### *Hdac4* Participates in Frontal Bone Development

**FIGURE 1:**
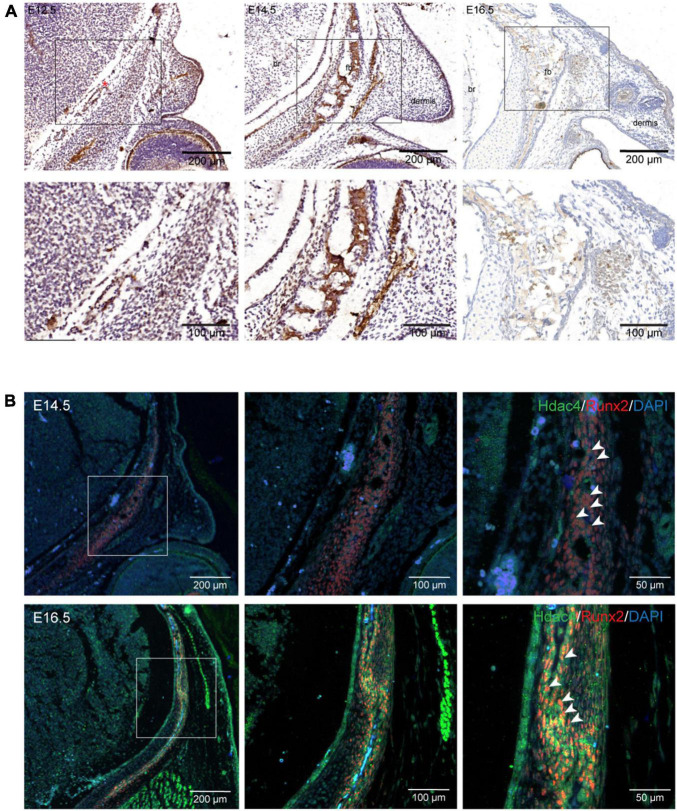
*Hdac4* expression during frontal bone development. **(A)** Immunohistochemical staining of coronal sections of the embryonic mouse head at E12.5, E14.5, and E16.5. At E12.5, HDAC4 expression was not evident in condensed mesenchymal cells in the presumptive frontal primordium (red asterisk). HDAC4 expression was detected in the frontal bone at E14.5 and E16.5. **(B)** Immunofluorescence staining of coronal sections of the embryonic mouse head at E14.5 and E16.5. HDAC4 expression largely overlapped with that of Runx2 (white arrowhead). Br, brain; fb, frontal bone.

To explore the role of *Hdac4* in frontal bone development, we first examined HDAC4 protein expression in the mouse embryonic head at different developmental stages. At E14.5 and E16.5, HDAC4 protein was strongly expressed in the frontal bone primordium, but could hardly be detected in condensed mesenchymal cells in the presumptive frontal primordium at E12.5 (Figure 1A). To confirm that Hdac4 was indeed expressed in presumptive osteoblasts, we examined the Runx2 expression domain by immunofluorescence in coronal sections from E14.5 and E16.5 embryos. As expected, the Runx2 expression domain overlapped with that of HDAC4 in the frontal primordium (Figure 1B). These results indicated that *Hdac4* might be involved in frontal bone development.

### Conditional Knockout of *Hdac4* in Cranial Neural Crest Cells Leads to Decreased Frontal Bone Formation

**FIGURE 2:**
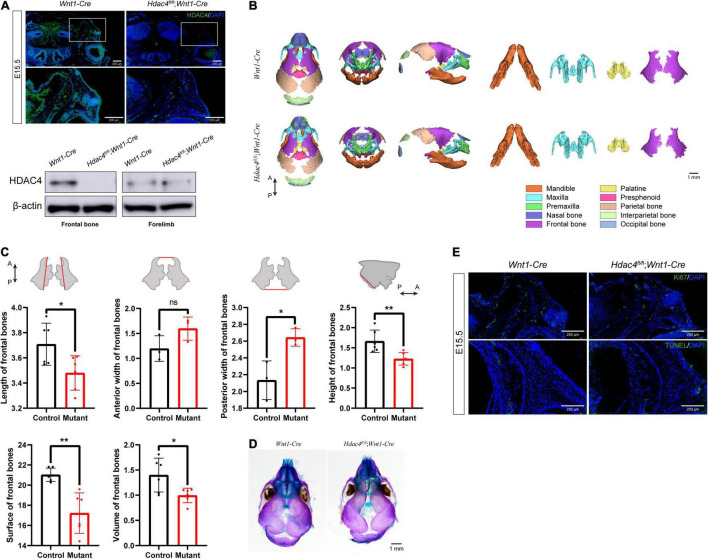
The conditional knockout of *Hdac4* in cranial neural crest cells (CNCCs) results in an underdeveloped frontal bone. **(A)** Immunofluorescence staining of HDAC4 in frontal bone at E15.5. Western blotting of frontal bone and forelimbs in control and *Hdac4*^fl/fl^*;Wnt1-Cre* mutant mice at P0. **(B)** Posteroanterior, lateral, and frontal views of micro-computed tomographic rendering of a skull of a P0 *Wnt1-Cre* and *Hdac4*^fl/fl^*;Wnt1-Cre* mutant mouse. Note the decreased frontal bone formation in the *Hdac4*^fl/fl^*;Wnt1-Cre* mutants. The maxilla, mandible, palatine bone, and frontal bone were isolated at P0. *Hdac4*^fl/fl^*;Wnt1-Cre* mutant mice exhibited smaller frontal bones and there was a wider interfrontal gap between frontal bones compared with that in *Wnt1-Cre* (control) mice. The palatine bones of *Hdac4*^fl/fl^*;Wnt1-Cre* mutant mice were slightly smaller than those of *Wnt1-Cre* mice, while the maxilla and mandibles were of normal size and had no macroscopic deformities. **(C)** Quantification of the size (length, width, and height) of frontal bones from control (*n* = 3) and *Hdac4*^fl/fl^*;Wnt1-Cre* mutant mice (*n* = 3). Comparison of the surface area and volume of frontal bones from control (*n* = 3) and *Hdac4*^fl/fl^*;Wnt1-Cre* mutant mice (*n* = 3). P→A: posterior to anterior. *P < 0.05; **P < 0.01; ns. not significant. **(D)** Skeletal preparations showing the top view of the frontal bones in the P0 control and *Hdac4*^fl/fl^*;Wnt1-Cre* mutant pups. **(E)** Immunofluorescence staining of Ki67 and TUNEL staining in the frontal bone at E15.5.

To determine the role of *Hdac4* in the development of the craniofacial skeleton, we generated mice with conditional knockout of *Hdac4* in CNCCs by crossing *Hdac4^fl/fl^* mice with *Wnt1-Cre* mice (Supplementary Figure 1). Immunofluorescence staining of coronal sections of the head of E15.5 embryos showed that HDAC4 expression was significantly reduced in *Hdac4*^fl/fl^*;Wnt1-Cre* mice compared with that in control mice. Using Western blot analysis, we confirmed that HDAC4 expression was specifically reduced in CNCC-derived craniofacial tissues, but not in the forelimb, of *Hdac4*^fl/fl^*;Wnt1-Cre* at P0 (Figure 2A). *Hdac4*^fl/fl^*;Wnt1-Cre* pups at birth exhibited normal craniofacial size, and no macroscopic deformities were apparent. However, volume rendering of micro-CT images showed that the frontal bones of *Hdac4*^fl/fl^*;Wnt1-Cre* pups were deformed and smaller than those of their control littermates (Figure 2B).

Next, we isolated and measured the size of the frontal bones of control and *Hdac4*^fl/fl^*;Wnt1-Cre* mice. To compare bone size, the bones were labeled with anatomical landmarks that could be used as reference points, as defined in a previous study (Garcia-Wilson and Perkins, 2005). The frontal bones were significantly smaller in *Hdac4*^fl/fl^*;Wnt1-Cre* mice than in control mice by approximately 85.7% in length, 142.7% in posterior width (a larger posterior gap between both frontal bones), and 74.2% in height; no significant differences were seen in anterior width. Bone surface and volume in *Hdac4*^fl/fl^*;Wnt1-Cre* mice were approximately 86.4 and 75.8%, respectively, those of control mice (Figure 2C). Additionally, *Hdac4*^fl/fl^*;Wnt1-Cre* mutant mice exhibited increased bone porosity and impaired frontal bone development, characterized by a wide interfrontal suture between the two frontal bones (Figure 2D).

Next, we sought to determine the cause of the reduced frontal bone formation in *Hdac4*^fl/fl^*;Wnt1-Cre* mice. We found that the expression of Ki67, a well-known proliferation marker, in coronal sections of the E15.5 head was significantly reduced in the frontal primordia of *Hdac4*^fl/fl^*;Wnt1-Cre* mice (Figure 2E), indicating that cell proliferation was suppressed in the frontal bone. To determine whether the disrupted frontal bone formation resulted from an increase in abnormal cell death, we performed a TUNEL assay on histological sections. The cell apoptosis signal of osteoblast lineages within the frontal primordia was indistinguishable between mutant and control mice (Figure 2E). These data suggested that *Hdac4* is involved in the proliferation, but not survival, of CNCC-derived osteoblast lineage cells during frontal bone development. The decreased proliferative potential of osteoblast lineage cells could explain the frontal bone defects in *Hdac4*^fl/fl^*;Wnt1-Cre* mice. Overall, these results indicated that the conditional knockout of *Hdac4* in CNCC-derived osteogenic lineage cells leads to decreased cell proliferation, which results in frontal bone defects in *Hdac4*^fl/fl^*;Wnt1-Cre* mice.

### Ablation of *Hdac4* Resulted in Decreased Osteoblast Proliferation

**FIGURE 3:**
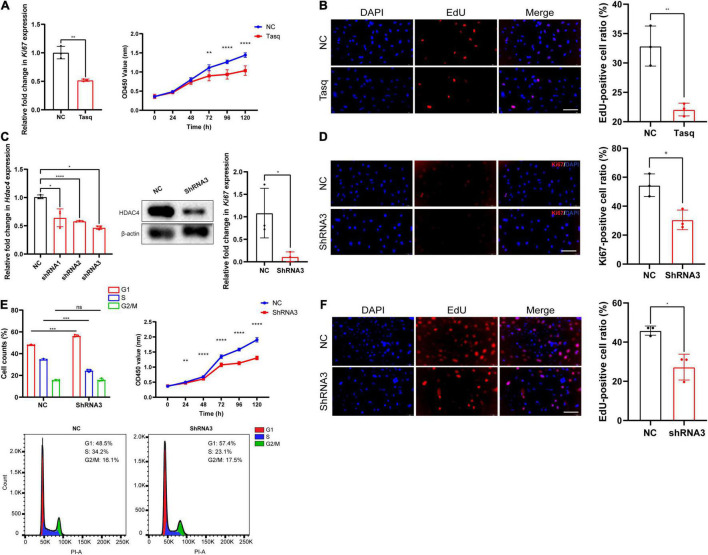
The effect of *Hdac4* on the proliferation of MC3T3-E1 cells. **(A)** RT-qPCR analysis of *Ki67* expression in cells treated with tasquinimod (Tasq) (10 μM) for 7 days. Cell proliferation assays were performed for control and tasquinimod-treated (10 μM) cells at 0, 24, 48, 72, 96, or 120 h (0–5 days). **(B)** Images and quantification of EdU staining (red) in MC3T3-E1 cells after treatment with 10 μM tasquinimod for 7 days. Nuclei were counterstained with DAPI (blue). Scale bar, 100 μm. **(C)** RT-qPCR analysis of *Hdac4* knockdown in MC3T3-E1 cells transfected with negative control (NC) or one of three Hdac4-specific shRNAs (*n* = 3). Western blotting of HDAC4 in control and shRNA3-transfected cells. RT-qPCR of *Ki67* in control and *Hdac4* knockdown cells. **(D)** Immunofluorescence staining of Ki67 in control and shRNA3-transfected cells. **(E)** Flow cytometric analysis of the effect of *Hdac4* knockdown on the cell cycle. The bar graph represents the percentage distribution of control and *Hdac4* knockdown cells in different phases of the cell cycle. The cell cycle distribution of control and *Hdac4* knockdown cells is shown. Cell proliferation assays were performed in control and *Hdac4* knockdown cells at 0, 24, 48, 72, 96, or 120 h after transfection (day 0–5). **(F)** Images and quantification of EdU staining (red) in control and *Hdac4* knockdown cells. Nuclei were counterstained with DAPI (blue). Scale bar, 100 μm. *P < 0.05; **P < 0.01; ***P < 0.001; ****P < 0.0001.

To clarify the potential role of *Hdac4* in the proliferation of pre-osteoblasts, we first performed cell proliferation assays on MC3T3-E1 cells treated either with the HDAC4 inhibitor tasquinimod (10 μM) or DMSO (10 μM, control). The optimum concentration of tasquinimod was determined based on preliminary experiments with the 1, 2.5, 5, and 10 μM concentrations. The HDAC inhibiting efficiency was verified by assessment of upregulation of *Runx2* expression using RT-qPCR (Supplementary Figure 2). The RT-qPCR results showed that, compared with the controls, *Ki67* expression was downregulated (0.5-fold) in tasquinimod-treated cells (Figure 3A). CCK-8 and EdU incorporation assays further showed that tasquinimod treatment at the 10 μM concentration inhibited the proliferative ability of MC3T3-E1 cells (Figures 3A,B).

To further explore the role of *Hdac4* in cell proliferation *in vitro*, we established *Hdac4* knockdown MC3T3-E1 cells through the transfection of shRNA expression vectors. The protein and mRNA levels of Hdac4 were subsequently detected by Western blotting and RT-qPCR, respectively (Figure 3C). We found that Hdac4-shRNA3 exerted the greatest inhibitory effect on the expression level of *Hdac4* (*p* < 0.0001). Consequently, Hdac4-shRNA3-expressing cells were used for further experiments. As shown in Figure 2C, *Ki67* mRNA expression was significantly downregulated in the Hdac4-shRNA3-expressing cells compared with that in cells treated with the negative control (Hdac4-NC) (*p* < 0.05). Immunofluorescence staining further showed that the numbers of Ki67-positive cells were significantly reduced in the Hdac4-shRNA3 treatment group (Figure 3D). The results of the CCK-8 assay demonstrated that the proliferation rate of Hdac4-shRNA3-treated cells was lower than that of control cells (Figure 3E). In addition, flow cytometric analysis showed that, compared with controls, the proportion of cells in the G1 phase was significantly increased (57.4%), whereas that of cells in the S phase was decreased (23.1%) in Hdac4-shRNA3-expressing cells (Figure 3E). EdU staining showed that the ratio of EdU-positive cells was significantly lower in the Hdac4-shRNA3 treatment group than in the negative control group (*p* < 0.05) (Figure 3F). Together, these data indicated that *Hdac4* facilitates cell proliferation in MC3T3-E1 cells.

### The Gene Expression Profile of MC3T3-E1 Cells Is Altered After *Hdac4* Knockdown

**FIGURE 4:**
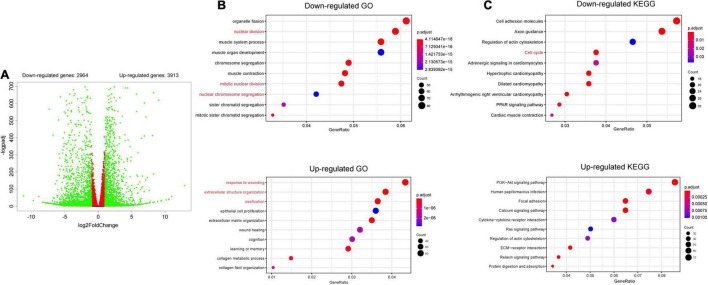
RNA sequencing of MC3T3-E1 cells transfected with scramble or shRNA3 lentivirus (*n* = 3). **(A)** Volcano plots for all the genes of the control and Hdac4-shRNA3 groups. Green dots on both sides indicate up- and downregulated differentially expressed genes (DEGs; *p*-adj < 0.05). **(B)** The top 10 GO terms associated with biological processes (*p*-adj < 0.05) involving the downregulated genes in the Hdac4-shRNA3 group. **(C)** The top 10 enriched KEGG pathways associated with the downregulated genes (*p*-adj < 0.05) in the Hdac4-shRNA3 group.

To investigate the potential mechanisms involved in how *Hdac4* regulates cell proliferation in MC3T3-E1 cells, cells transfected with scramble or Hdac4-shRNA3 lentivirus (three biological replicates) were subjected to RNA sequencing. Analysis of the differentially expressed genes (DEGs; log2FC > 0.05, *p*-adj < 0.05) revealed that 3,913 genes were upregulated and 2,964 downregulated in Hdac4-shRNA3-expressing cells compared with that in control cells (Figure 4A). Gene Ontology (GO) analysis of all the DEGs showed that the downregulation of *Hdac4* suppressed cell proliferation through the regulation of biological processes associated with “mitotic nuclear division,” “nuclear division,” and “chromosome segregation” (Figure 4B), and promoted biological process such as “respond to wound healing,” “extracellular matrix organization,” and “ossification,” which is in line with previously reported results (Vega et al., 2004). The results of GO enrichment analysis indicated that multiple biological processes related to cell proliferation were affected in the Hdac4-shRNA3 group. Furthermore, “PI3K-Akt signaling pathway,” “calcium signaling pathway,” and “extracellular matrix-receptor (ECM-receptor) interaction pathway” were among the most enriched pathways among the upregulated genes. The pathways enriched among the downregulated genes in the Hdac4-shRNA3 group included “cell cycle,” which is closely related to cell proliferation (Figure 4C).

### *Hdac4* Promotes Osteoblast Proliferation by Regulating the Expression of Multiple Cell Cycle-Related Genes

**FIGURE 5:**
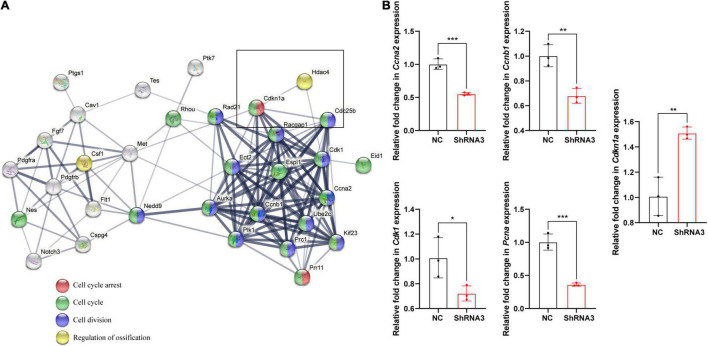
**(A)** The STRING database (https://string-db.org/) was used to analyze the protein–protein interaction network relating to the cell cycle-related genes present among the top 500 differentially expressed genes and their interaction with Hdac4. Protein interactions between HDAC4 and CDKN1A as determined by STRING-based analysis. **(B)** RT-qPCR analysis of cell cycle-related genes in control and Hdac4-shRNA3-transfected cells. *P < 0.05; **P < 0.01; ***P < 0.001.

**FIGURE 6:**
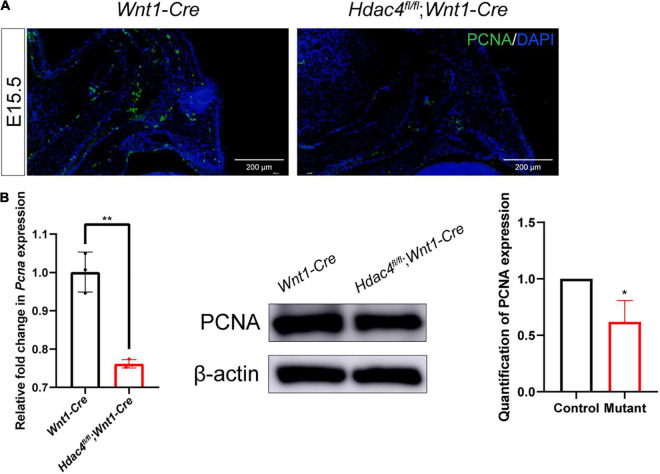
**(A)** Immunofluorescence staining of PCNA in the frontal bone of control and *Hdac4*^fl/fl^*;Wnt1-Cre* mutant mice at P0. **(B)** RT-qPCR analysis and western blotting of PCNA in the frontal bone of control and *Hdac4*^fl/fl^*;Wnt1-Cre* mutant mice. *P < 0.05; **P < 0.01.

STRING analysis showed that *Hdac4* knockdown resulted in changes in the expression of multiple cell cycle-related genes (Figure 5A). Among the identified differentially expressed cell cycle-related genes was *Cdkn1a*, a negative regulator of the G1/S-phase of the cell cycle. STRING analysis indicated that HDAC4 interacted with CDK1 and CDKN1A. We also validated the mRNA expression of several cell cycle-related genes in *Hdac4* knockdown MC3T3-E1 cells. As expected, the mRNA expression of *Cdk1*, *Ccna2*, *Ccnb1*, and *Pcna* was significantly decreased, whereas that of *Cdkn1a* was significantly increased in Hdac4-shRNA3-expressing cells (Figure 5B). These data are in accordance with the phenotypes reported above and demonstrate that *Hdac4* downregulation may suppress cell proliferation through its negative effects on the expression of multiple cell cycle-related genes in MC3T3-E1 cells. Next, we focused on *Pcna*, which defines the late G1/S and early G2/M subpopulations of the cell cycle, to determine whether the downregulation of *Pcna* might contribute to the observed frontal bone defects. We found that the expression of *Pcna* was downregulated in the E15.5 embryonic head (Figure 6A). Western blot analysis of the frontal bone in control and *Hdac4*^fl/fl^*;Wnt1-Cre* pups further confirmed the downregulation of *Pcna* (Figure 6B), indicating that this gene might be a downstream target of *Hdac4* in the regulation of cell proliferation in the developing frontal bone. These results suggested that decreased cell proliferation and frontal bone defects due to *Hdac4* deficiency may be attributable, at least part, to the resulting negative effects on *Pcna* expression.

## Discussion

HDAC4, a class II HDAC, plays a critical role in numerous biological processes, including cell differentiation and proliferation, by regulating gene transcription. Although HDAC inhibitors are commonly used in clinical settings (Duvic and Vu, 2007), babies born from HDAC inhibitor-treated mothers show abnormal craniofacial development, highlighting the critical need to unravel the role that HDACs play during head development. Studies have revealed that the class I HDACs, *Hdac3* and *Hdac8*, are required for proper craniofacial development. The deletion of *Hdac3* and *Hdac8* in CNCCs results in various craniofacial deformities, including cleft palate, microcephaly, and calvarial bone defects (Haberland et al., 2009; Singh et al., 2013). *Hdac4*-null mice exhibit ectopic chondrocyte hypertrophy during endochondral ossification. The forelimb, ribcage, vertebrae, and base of the skull, but not the rest of the craniofacial bones, are severely affected in *Hdac4*-null mice (Vega et al., 2004). DeLaurier et al. revealed mRNA expression of *hdac4* in migrating CNCCs in the head at 15 hpf (hours post fertilization) in zebrafish. MO-knockdown of *hdac4* in zebrafish led to a severe reduction of migratory CNCCs in early embryogenesis, which resulted in facial shortening and defects in palatal skeleton cartilage (DeLaurier et al., 2012, 2019).

In the present study, using tissue-specific approaches, we provided evidence for the first time that CNCC-intrinsic *Hdac4* activity is required for proper frontal bone development in the mouse. Our data showed that *Hdac4* is hardly expressed in the early stage of embryonic head at E10.5 and E11.5. *Hdac4* expression was first detected in osteogenic fronts and craniofacial bone primordia at E14.5, that is, when craniofacial bone formation begins. These results indicated that, in contrast to the function of *hdac4* in migratory CNCCs in zebrafish, *Hdac4* in mice mainly functions in later stage of embryonic craniofacial development, when CNCCs differentiate into osteoblasts. *Hdac4* deficiency in CNCCs of *Hdac4*^fl/fl^*;Wnt1-Cre* pups at P0 led to defects in multiple craniofacial bones, particularly the frontal bones. In *Hdac4*^fl/fl^*;Wnt1-Cre* mice, the frontal bones exhibited decreased length, height, and posterior width, but not anterior width. The size and the volume of the frontal bones were smaller in *Hdac4*^fl/fl^*;Wnt1-Cre* mice than in the controls. However, maxilla and mandible size was not affected. Although *Hdac4* was also knocked out in other craniofacial structures, including the maxilla and mandibles, no significant defects were observed (Supplementary Figure 3). These findings indicate that different craniofacial structures have different sensitivities to HDAC inhibition. *Hdac4* knockdown significantly reduced the proliferative activity of MC3T3-E1 cells by arresting the cell cycle at the G1 phase, similar to its previously described role in other cell types, such as skeletal muscle satellite cells and vascular smooth muscle cells (Wang et al., 1999; Cao et al., 2019). In accordance with the *in vitro* findings, *Hdac4*^fl/fl^*;Wnt1-Cre* pups showed reduced cell proliferation in CNCC-derived osteoblasts in the frontal bone. Collectively, these observations indicate that *Hdac4* expression positively regulates osteoblast proliferation.

We found that the suppression of osteoblast proliferation by *Hdac4* ablation was attributable to its negative effects on the expression of cell cycle-related genes, including *Cdkn1a*, *Ccna2*, *Ccnb1*, *Cdk1*, and *Pcna*. *Hdac4* knockdown led to the upregulation of negative regulators of the cell cycle, such as *Cdkn1a.* Studies have shown that HDAC4 represses *CDKN1A* expression in human cancer cells in a SP1-dependent manner (Qian et al., 2006). CDKN1A was reported to interact with multiple HDACs, including HDAC4, in a human osteosarcoma cell line (Seo et al., 2009). Notably, some genes that positively regulate cell proliferation, such as *Pcna*, were significantly downregulated following the knockdown of *Hdac4*. PCNA plays a critical role in cell proliferation initiation. In osteosarcoma cells, HDAC4 promotes cell proliferation through the regulation of *PCNA* expression (Geng et al., 2011). Although *Hdac4* mainly exerts its regulatory functions by deacetylating histones, and thereby exerting an inhibitory effect on transcription, *Hdac4* can also deacetylate some non-histone substrates to regulate their functions and stability. For instance, Wang et al. (1999) demonstrated that HDAC4 binds to the N terminus of MEF2C and subsequently represses its transcriptional activity (Choi et al., 2012). Recent studies have also implicated HDAC4 in the deacetylation of other non-histone proteins, such as HIF1α, MEKK2, and STAT1 (Lichtenstein et al., 1972; Pratap et al., 2003; Thomas et al., 2004; Stronach et al., 2011; Zhou et al., 2013; Liu et al., 2019). These observations indicate that the abrogation of *Hdac4* activity can induce profound changes in gene expression programs in osteoblasts both directly and indirectly. More studies are needed to further clarify the mechanism underlying how *Hdac4* regulates the expression of cell cycle-related genes during craniofacial development.

HDAC4 has been reported to regulate endochondral bone formation by interacting with Runx2 and inhibiting its activity (Vega et al., 2004). In line with previous studies, our RNA-seq results demonstrated that *Hdac4* knockdown led to an increase in the expression of osteogenesis-related genes, including *Runx2*, *Col1a1*, and *Sp7*. Surprisingly, however, *Hdac4*^fl/fl^*;Wnt1-Cre* mice exhibited reduced frontal bone formation. Several studies have shown that Runx2 exerts an inhibitory effect on cell proliferation (Liu et al., 2013; Xu et al., 2019). Pratap et al. (2003) demonstrated that Runx2 not only promotes bone maturation, but also has a role in attenuating osteoblast growth in osteogenic lineage cells (Xu et al., 2019). Meanwhile, *Hdac4* deficiency in mouse osteoblasts leads to smaller stature along with decreased bone formation (Nakatani et al., 2018). These findings indicate that the upregulation of *Runx2* expression following *Hdac4* depletion might result in reduced osteoblast proliferation and increased ossification, thereby contributing to frontal bone defects in *Hdac4*^fl/fl^*;Wnt1-Cre* mice.

## Conclusion

In conclusion, our findings suggested that craniofacial skeletal development is sensitive to Hdac4 activity. We also found that reduced proliferation of craniofacial osteoblast lineage cells contributed to the frontal bone defects observed in *Hdac4*^fl/fl^*;Wnt1-Cre* mice. Genome-wide transcriptome profiling in osteoblasts further revealed that HDAC4 regulates a large number of genes involved in skeletal development and proliferation, providing important information for future genetic dissection of the regulatory network underlying craniofacial skeletal development. Our *Hdac4* conditional knockout model provides an excellent system for dissecting the contributions of epigenetic factors to the intricate process of craniofacial morphogenesis.

## Data Availability Statement

The datasets presented in this study can be found in online repositories. The names of the repository/repositories and accession number(s) can be found below: NCBI GEO GSE186707.

## Ethics Statement

The animal study was reviewed and approved by the Animal Experimental Ethical Inspection of the Shanghai Ninth People’s Hospital Affiliated to Shanghai Jiao Tong University, School of Medicine.

## Author Contributions

NH and JS conceived, designed, and performed the experiments and analyzed the data. QB, DW, and XW contributed to the interpretation of the results and critical revision of the manuscript. All authors agreed to be accountable for the content of this work.

## Conflict of Interest

The authors declare that the research was conducted in the absence of any commercial or financial relationships that could be construed as a potential conflict of interest.

## Publisher’s Note

All claims expressed in this article are solely those of the authors and do not necessarily represent those of their affiliated organizations, or those of the publisher, the editors and the reviewers. Any product that may be evaluated in this article, or claim that may be made by its manufacturer, is not guaranteed or endorsed by the publisher.
