# Supplementary material for: Hdac4 Regulates the Proliferation of Neural Crest-Derived Osteoblasts During Murine Craniofacial Development
Source: Front Physiol. 2022 Feb 15;13:819619. doi: 10.3389/fphys.2022.819619 (PMC8886889; doi:10.3389/fphys.2022.819619)

Supplementary Material

# Supplementary Figures


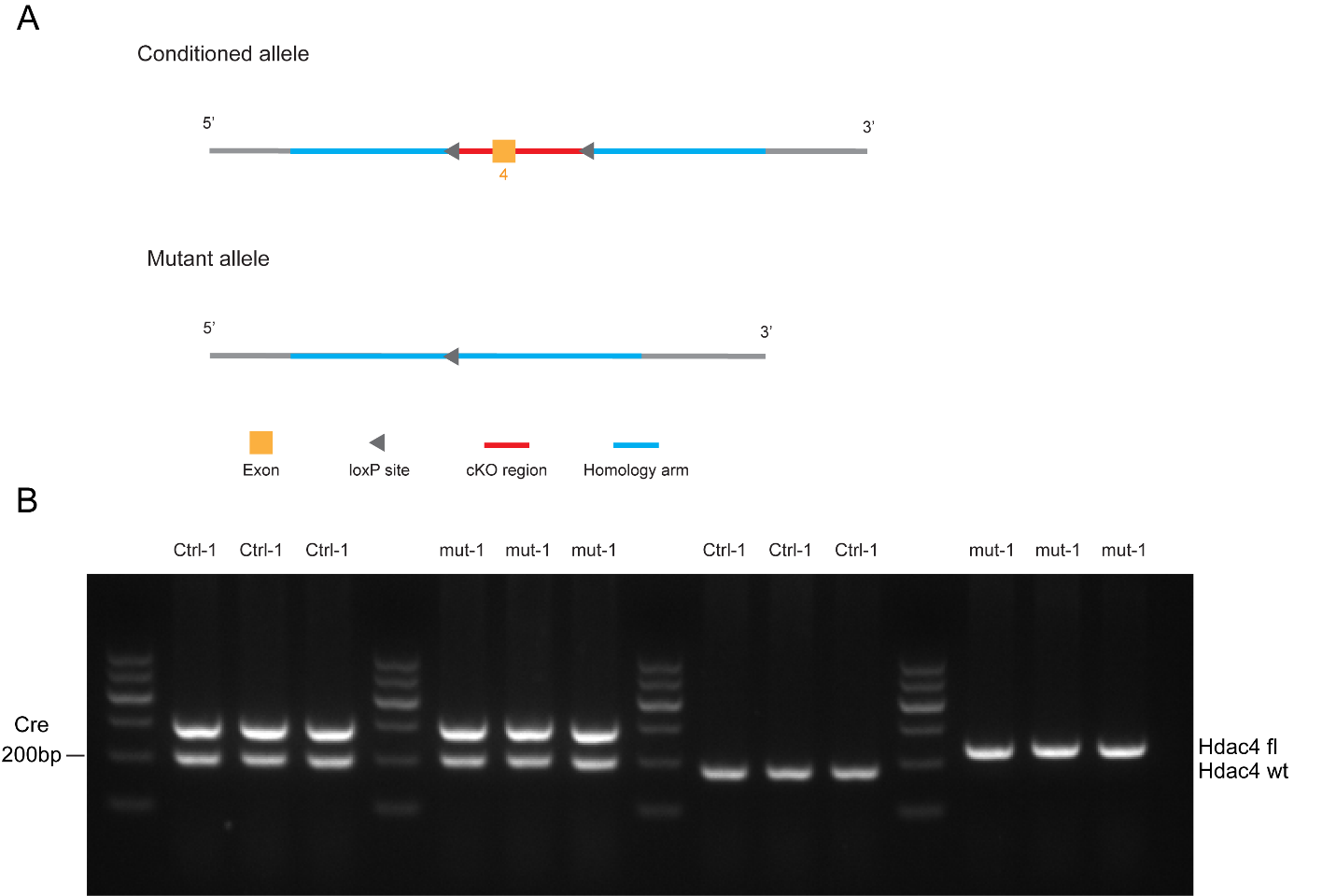
**Supplementary Figure 1.** (**A**) Schematic diagram of the generation of *Hdac4* conditional knockout mice. (**B**) PCR genotyping of *Wnt1-Cre* (control [Ctrl], *n*=3) and *Hdac4^fl/fl^;Wnt1-Cre* (mutant [mut], *n*=3) mice used for micro-CT imaging.


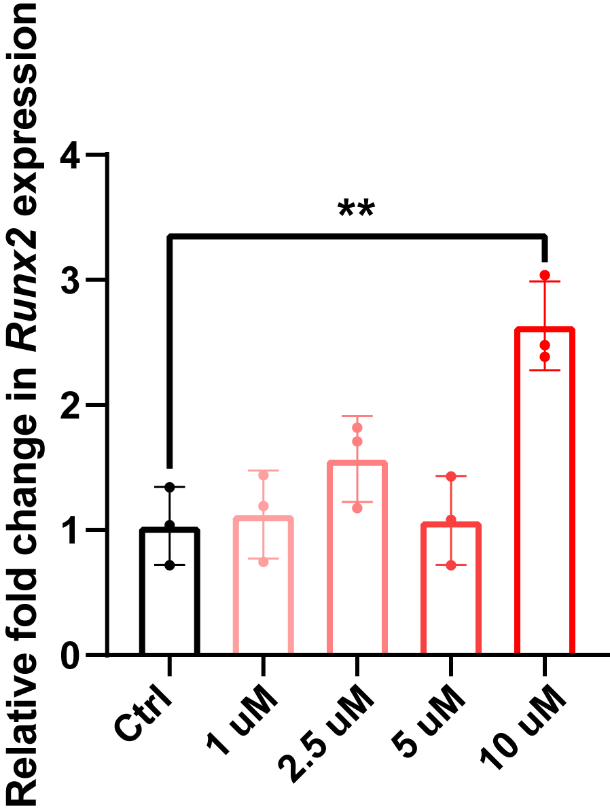


**Supplementary Figure 2.** Determination of the optimum cencentration of tasquinomod. RT-qPCR analysis of *Runx2* expression in control (Ctrl) and MC3T3 cells treated with different concentration of tasquinimod (1 µM, 2.5 µM, 5 µM, and 10 µM). **P<0.01.


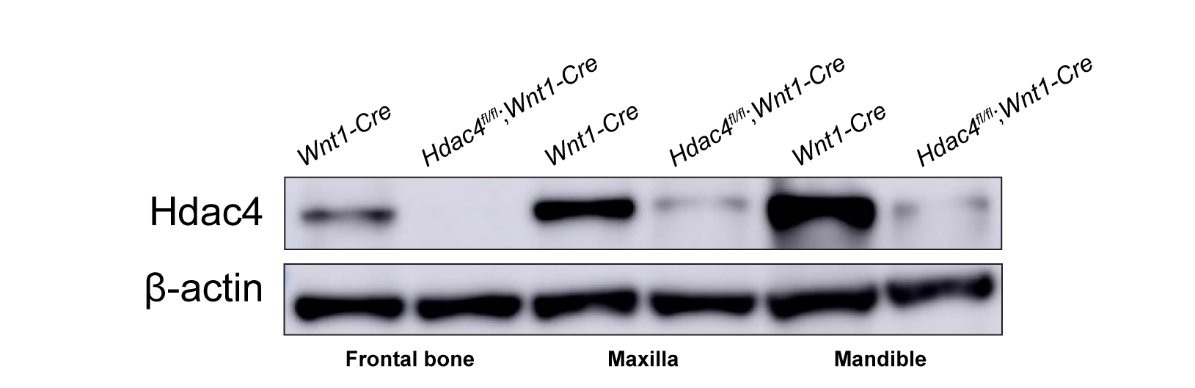


**Supplementary Figure 3.** Western blotting of the frontal bone, maxilla, and mandible of control mice and *Hdac4^fl/fl^*;*Wnt1-Cre* mutants.

# Supplementary Tables


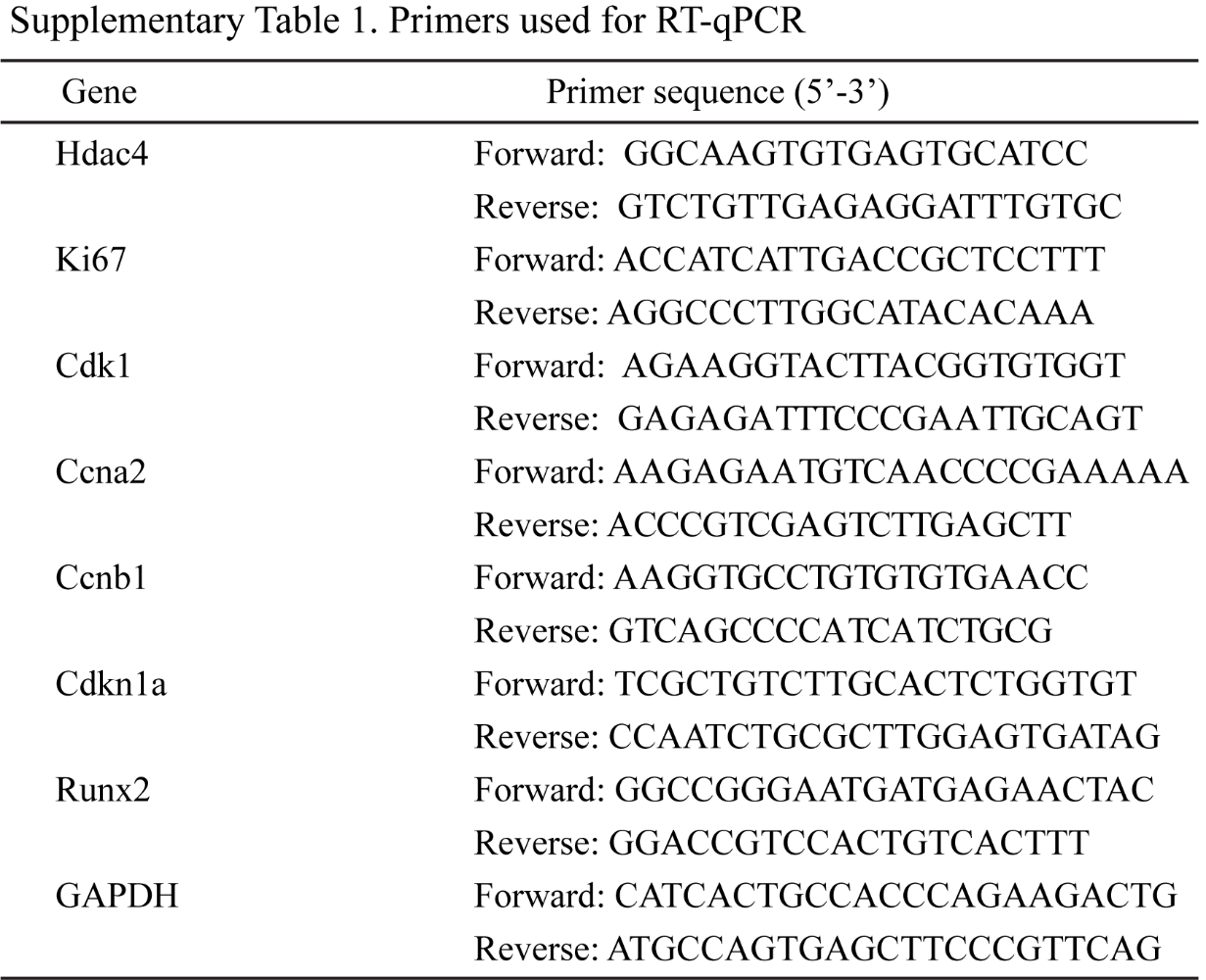

Supplement: Supplementary file 1 [file Data_Sheet_1.docx]
